# Intrathecal Administration of Morphine Decreases Persistent Pain after Cesarean Section: A Prospective Observational Study

**DOI:** 10.1371/journal.pone.0155114

**Published:** 2016-05-10

**Authors:** Kumi Moriyama, Yuki Ohashi, Akira Motoyasu, Tadao Ando, Kiyoshi Moriyama, Tomoko Yorozu

**Affiliations:** Department of Anesthesiology, Kyorin University School of Medicine, 6-20-2 Shinkawa, Mitaka, Tokyo, 181–8611, Japan; The University of Tokyo Hospital, JAPAN

## Abstract

**Purpose:**

Chronic pain after cesarean section (CS) is a serious concern, as it can result in functional disability. We evaluated the prevalence of chronic pain after CS prospectively at a single institution in Japan. We also analyzed perioperative risk factors associated with chronic pain using logistic regression analyses with a backward-stepwise procedure.

**Materials and Methods:**

Patients who underwent elective or emergency CS between May 2012 and May 2014 were recruited. Maternal demographics as well as details of surgery and anesthesia were recorded. An anesthesiologist visited the patients on postoperative day (POD) 1 and 2, and assessed their pain with the Prince Henry Pain Scale. To evaluate the prevalence of chronic pain, we contacted patients by sending a questionnaire 3 months post-CS.

**Results:**

Among 225 patients who questionnaires, 69 (30.7%) of patients complained of persistent pain, although no patient required pain medication. Multivariate analyses identified lighter weight (p = 0.011) and non-intrathecal administration of morphine (p = 0.023) as determinant factors associated with persistent pain at 3 months. The adjusted odds ratio of intrathecal administration of morphine to reduce persistent pain was 0.424, suggesting that intrathecal administration of morphine could decrease chronic pain by 50%. In addition, 51.6% of patients had abnormal wound sensation, suggesting the development of neuropathic pain. Also, 6% of patients with abnormal wound sensation required medication, yet no patients with persistent pain required medication.

**Conclusion:**

Although no effect on acute pain was observed, intrathecal administration of morphine significantly decreased chronic pain after CS.

## Introduction

Relieving postoperative pain is especially beneficial for a woman having a cesarean section, since uncontrolled pain can impair the ability to care for her baby in the immediate *post partum* period [1]. Severe acute pain after cesarean section is associated with persistent pain and depression 8 weeks after delivery as well [2]. Chronic pain can be associated with negative long-term effects on a mother, and may result in functional disability [3]. Due to the increasing number of cesarean sections being carried out [4], relieving post-cesarean pain has become increasingly important.

Studies have elucidated the risk factors associated with the development of chronic pain after cesarean delivery: poorly controlled acute pain [2, 5], type of anesthetic given [3] and previous delivery [6]. However, little is known about the prevalence and risk factors of chronic pain after cesarean section in Japan. Genetic, cultural and perioperative factors affect pain perception [7], so identification of the risk factors for chronic postoperative pain in each country is important.

We wished to investigate prospectively the incidence of chronic pain after cesarean section in a single institution in Tokyo, Japan. We also analyzed the type of perioperative risk factors associated with chronic pain.

## Materials & Methods

### Study Design and Data Collection

This prospective observational study was conducted at Kyorin University Hospital (Tokyo, Japan). The study protocol was approved by our Institutional Review Board on Human Research (approval number H23-153-03). Written informed consent was obtained from all patients. The study period was between May 2012 and May 2014. Patients who were scheduled for elective cesarean section during the study period were recruited into the study. Maternal demographics (age, height, body weight, parity, systemic illness, reason for cesarean delivery, and history of related factors) were collected.

Spinal, combined spinal–epidural or general anesthesia were chosen at the discretion of the attending anesthesiologist. For spinal anesthesia, a local anesthetic (12mg bupivacaine) with 10 μg fentanyl was injected into the subarachnoid space with or without 0.1 mg of morphine. An intravenous sedative (diazepam or midazolam) and/or an opioid (fentanyl), and/or epidural local anesthetic were given during surgery at the discretion of the attending anesthesiologist. Patients who did not have morphine *via* the intrathecal route received a continuous infusion of epidural ropivacaine or subcutaneous fentanyl as postoperative analgesia. Details of surgery and anesthesia, such as the type of anesthesia (regional or general), procedure type (elective or emergency), duration of surgery and type of incision (vertical or transverse) were also recorded.

Postoperatively, patients received pentazocine (30 mg i.v.), flurbiprofen (50 mg, i.v.), loxoprofen (60 mg, p.o.), or acetaminophen (400 mg, p.o.) if they complained of pain. We routinely monitored hourly respiratory rate, consciousness, continuous oxygen saturation (SpO_2_) and pulse rate as determined by a pulse oximeter. These parameters were recorded by nurses until 9 am the next morning. Nurses were educated about respiratory depression with morphine (i.t.) and instructed to call an anesthesiologist if the respiratory rate reached ≤10 breaths/min, or if SpO_2_ reached ≤95%. Prevalence of maternal adverse reactions (sedation, pruritus, nausea, and vomiting) and use of supplemental analgesics and medication for adverse reactions were gathered from nursing records. An anesthesiologist visited patients on postoperative day (POD) 1 and 2, and assessed pain with the Prince Henry Pain Scale (PHPS) [8].

To evaluate the prevalence of chronic pain, we contacted patients by sending a questionnaire 3 months after cesarean section. In the questionnaire, we asked if they were experiencing pain and, if so, to detail its frequency, intensity and type (Table 1). The questionnaire also addressed the effect of pain on daily activities, sleep and mood. We asked for the type of analgesia required and any visit to a doctor for pain management. Exclusively, we asked if patients were experiencing abnormal sensations in the operated area.

**Table 1:** Questions about pain after cesarean section.

1. Do you breast-feed your child?
2. Do you still have pain in the operated area?
If you still have pain in the operated area, please answer the following questions:
(a) Please rate your pain from 0 to 10 (0: no pain, 10: worst pain imaginable)
(b) Does the pain limit your daily function?
(c) Have you taken any medication because of pain in the operated area?
3. Do you feel abnormal sensations in the operated area?
If you feel an abnormal sensation in the operated area, please answer the following questions:
(a) Please rate your abnormal sensation from 0 to 10 (0: no pain, 10: worst pain imaginable)
(b) Does the abnormal wound sensation limit your daily function?
(c) Have you taken any medication because of the abnormal wound sensation?

### Statistical Analyses

Data are the mean ± standard deviation (SD) and median ± interquartile range (IQR) for normally and non-normally distributed continuous data, respectively. Unpaired *t*-tests and Wilcoxon rank sum tests were used for normally and non-normally distributed continuous data, respectively.

For multivariate analyses of factors associated with persistent pain at 3 months, logistic regression analyses with a backward-stepwise procedure were used. Covariates were selected based on clinical knowledge and previous studies, and were kept to multivariate analyses if p<0.1 was observed by univariate analyses, as with the study by McCabe et al [9]. During multivariate analyses, covariates were retained if they were found to change the model in a significant manner (p <0.05). The statistical output is reported as an odds ratio (OR) and a 95% confidence interval (CI).

All statistical analyses were done with EZR (Saitama Medical Center, Jichi Medical University, Omiya, Japan), which is a graphical user interface for R (R Foundation for Statistical Computing, v3.1.1) [10]. Levels of significance were set at p = 0.05.

## Results

We recruited 280 patients during the study period, and 225 letters (80.4%) were returned. Maternal demographics as well as details of their surgery and anesthesia are shown in Table 2. Twenty-five patients underwent emergency cesarean section after hospital admission. A total of 50.7% of patients had repeat cesarean delivery.

**Table 2:** Maternal demographics and details of surgery and anesthesia (n = 225).

Age (years)	Mean (SD)	35.4 (4.5)
Height (cm)	Mean (SD)	158.3 (5.3)
Body weight before cesarean section(kg)	Mean (SD)	62.9 (9.2)
Body mass index (kg/m^2^)	Mean (SD)	25.1 (3.4)
Gestational age (weeks)	Mean (SD)	37.0 (0.8)
Parity	Median (IQR)	1 (0–1)
Duration of surgery (min)	Mean (SD)	66.7 (27.1)
Repeated cesarean delivery	No. (%)	114 (50.7)
Emergency	No. (%)	25 (11.1)
Intrathecal injection of morphine	No. (%)	188 (83.6)
Epidural analgesia	No. (%)	20 (8.9)
General anesthesia	No. (%)	9 (4.0)
Spinal anesthesia only	No. (%)	198 (88.0)
Vertical incision	No. (%)	137 (60.9)

SD, standard deviation; IQR, interquartile range.

**Fig 1:**
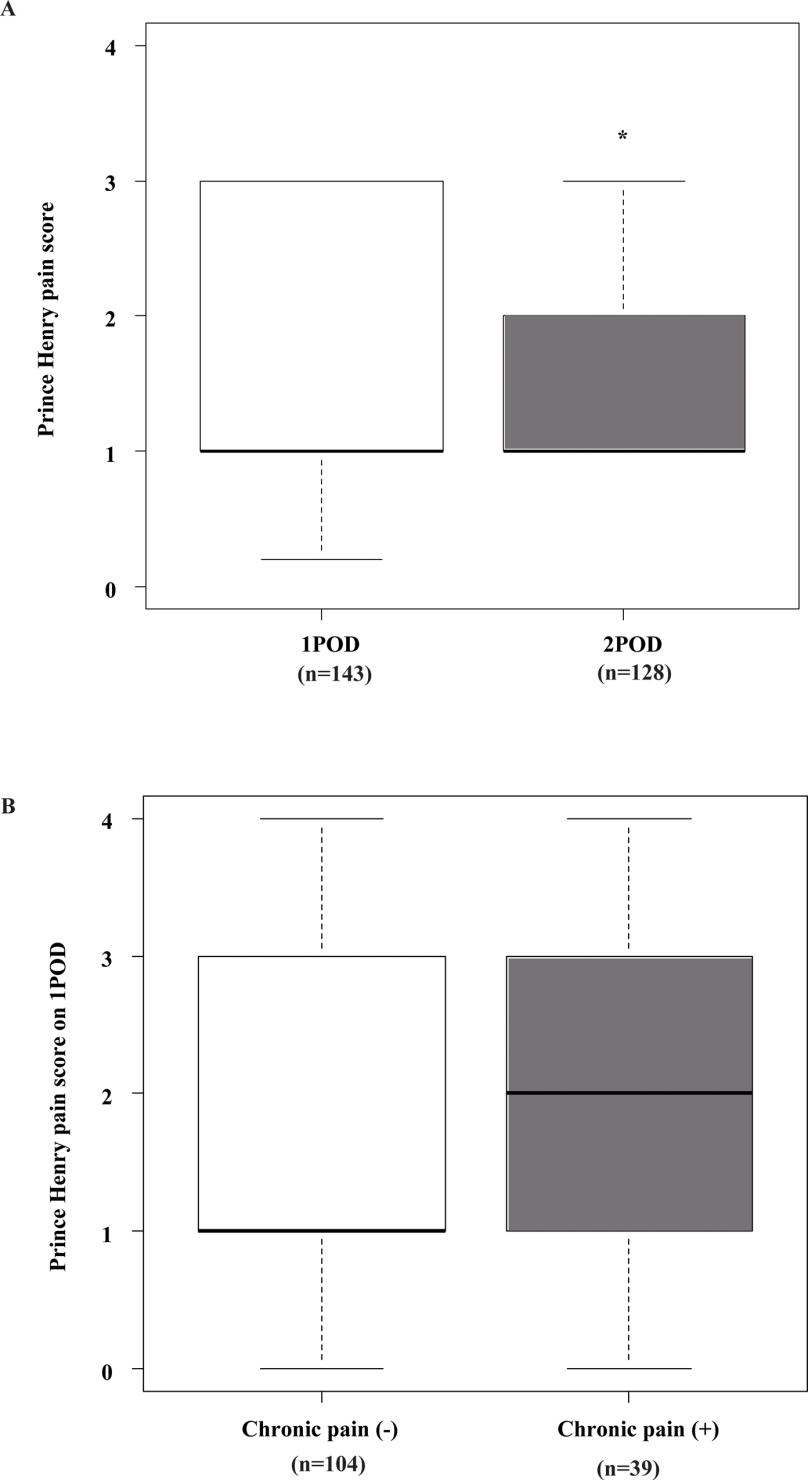
**(A) Pain severity on postoperative day (POD) 1 and 2 of cesarean delivery as assessed by the Prince Henry Pain Scale.** Pain severity was assessed by anesthesiologists who visited the patient, and was recorded with the PHPS: 1, no pain upon coughing; 2, pain upon coughing or movement but not on deep breathing; 3, pain on deep breathing but not at rest; 4, slight pain at rest; 5, severe pain at rest. *Indicates significantly lower PHPS score on POD2 compared with the PHPS score on POD1 by Wilcoxon rank sum tests (p < 0.001). **(B) Pain severity on POD1 in patients with and without chronic pain 3 months after cesarean delivery.** Pain severity on POD1 assessed by the PHPS was compared in patients with or without chronic pain 3 months after cesarean delivery. No significant difference was detected by Wilcoxon rank sum tests (p = 0.294).

Pain severity on POD1 and 2 is shown in Fig 1A. Median values of the PHPS were 1 on both POD1 and 2. On POD2, it decreased significantly from that on POD1 (p<0.001). We compared pain severity on POD1 among patients with or without persistent pain 3 months after delivery (Fig 1B). Although the median value of the PHPS on POD1 was 2 in patients with persistent pain, there was no significant difference (p = 0.294).

Table 3 summarizes the results of questionnaire-based interviews regarding persistent pain 3 months after delivery. Among 225 patients, only 1 patient did not answer whether she had any abnormal wound sensation. Although 30.7% of patients complained of any type of persistent pain, no patient required medication. More patients (51.6%) had abnormal wound sensation, and 6% of these patients required medication.

**Table 3:** Any persistent pain 3 months after delivery.

Patients with any persistent pain	69 (30.7%)
Patients whose pain limited daily function	10 (4.4%)
Patients who required pain medication	0 (0%)
Patients with any abnormal wound sensation	116 (51.8%)
Patients whose abnormal wound sensation limited daily function	7 (3.1%)
Patients who required medication for abnormal wound sensation	7 (3.1%)

Table 4 shows the association between persistent pain and abnormal wound sensations at 3 months after delivery. We found that 20.5% of patients had both persistent pain and abnormal wound sensations. Patients with persistent pain had significantly increased risk of abnormal wound sensations (p = 0.0029).

**Table 4:** Association between persistent pain and abnormal wound sensation 3 months after delivery.

		Patients with persistent pain		p
		(–)	(+)	
Patients with abnormal wound sensation	(-)	85	23	
	(+)	70	46	0.0029

Table 5 shows maternal demographics as well as details of surgery and anesthesia for patients with and without persistent pain at 3 months. Patients with persistent pain at 3 months were lighter (p = 0.0123); had a lower BMI (p = 0.0422); tended to be emergency cases (p = 0.0465); did not have morphine (i.t.) (p = 0.0275); had general anesthesia (p = 0.0388).

**Table 5:** Maternal demographics and details of surgery and anesthesia in patients with or without persistent pain at 3 months.

Variable		Persistent pain (+)	Persistent pain (–)	p
		(n = 69)	(n = 156)	
Maternal demographics				
Age (years)	Mean (SD)	35.0 (4.5)	35.6 (4.5)	0.3
Height (cm)	Mean (SD)	157.7 (5.9)	158.6 (5.0)	0.244
Body weight (kg)	Mean (SD)	60.7 (7.1)	63.9 (9.8)	0.0123
Body mass index (kg/m^2^)	Mean (SD)	24.4 (2.8)	25.4 (3.6)	0.0422
Parity	Median (IQR)	1 (1)	1 (1)	0.476
Repeated cesarean delivery	(%)	44.9	53.5	0.235
Gestational age (weeks)	Mean (SD)	37.0 (0.8)	36.9 (0.8)	0.493
Details of surgery and anesthesia				
Duration of surgery (min)	Mean (SD)	66.2 (19.4)	66.9 (30.0)	0.863
Emergency (%)	No. (%)	12 (17.4)	13 (8.3)	0.0465
Intrathecal injection of morphine (%)	No. (%)	52 (75.3)	136 (87.1)	0.0275
Epidural analgesia	No. (%)	7 (10.1)	13 (8.3)	0.661
General anesthesia	No. (%)	5 (7.2)	4 (2.6)	0.099
Vertical incision	No. (%)	46 (66.7)	91 (58.3)	0.261
Maternal adverse reactions				
Pain at rest on POD1	(%)	12.8	5.8	0.161
on POD2	(%)	2.9	3.2	0.916
Nausea on POD1	(%)	24.1	16.1	0.292
Vomiting on POD1	(%)	9.4	13.6	0.581
Pruritus on POD1	(%)	25.5	35.3	0.248
Sedation on POD1	(%)	22.4	28.4	0.429

SD, standard deviation; IQR, interquartile range; POD, postoperative day.

**Fig 2:**
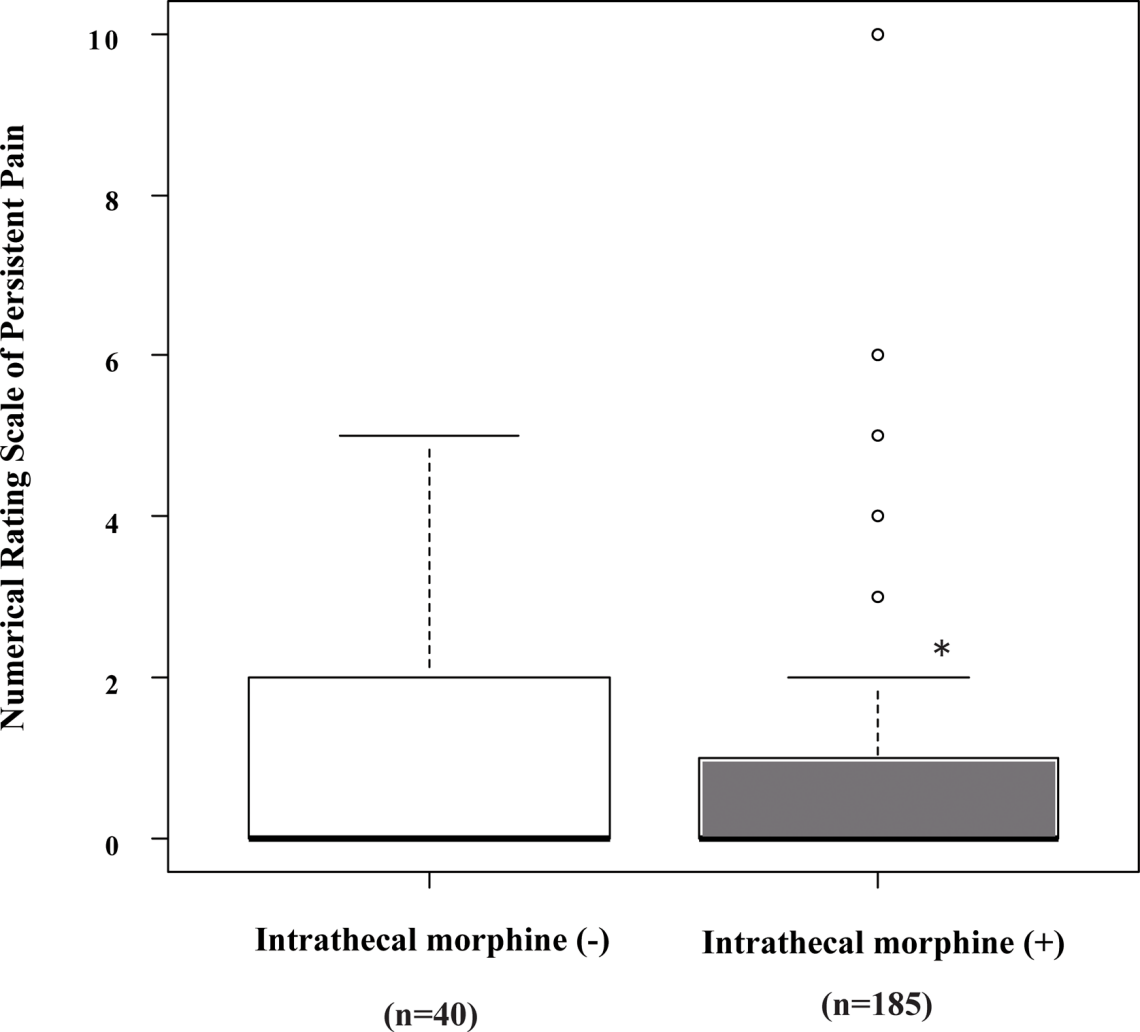
Pain severity at 3 month following cesarean delivery as assessed by the Numerical Rating Scale. The prevalence of persistent pain was evaluated by sending patients a questionnaire 3 months after cesarean section. *Indicates significantly lower NRS among patients with intrathecal administration of morphine by Wilcoxon rank sum tests (p<0.05).

Fig 2 shows the severity of persistent pain at 3 month after delivery, as assessed by the numerical rating scale (NRS) among patients with or without intrathecal administration of morphine. Median values of the NRS were 0. Pain severity at 3 month decreased significantly with intrathecal administration of morphine (p = 0.026). We excluded patients who had general anesthesia, and also found that intrathecal injection of morphine significantly decreased the severity of persistent pain (p = 0.0276).

Patients with abnormal wound sensations comprised more than half of all patients, so the sensations after delivery seemed to be as serious as persistent pain. Table 6 shows maternal demographics as well as details of surgery and anesthesia for patients with and without abnormal wound sensations at 3 months. We found no factor that significantly affected abnormal wound sensations at that point.

**Table 6:** Maternal demographics and details of surgery and anesthesia in patients with or without abnormal wound sensations at 3 months.

Variable		Abnormal wound sensations		p
		(+)	(-)	
	n	116	108	
Maternal demographics				
Age (years)	Mean (SD)	35.6 (4.3)	35.3 (4.6)	0.602
Height (cm)	Mean (SD)	158.2 (5.3)	158.5 (5.3)	0.607
Body weight (kg)	Mean (SD)	62.9 (8.7)	62.8 (9.7)	0.946
Body mass index (kg/m^2^)	Mean (SD)	25.1 (3.3)	25.0 (3.6)	0.764
Parity	Median (IQR)	1 (1)	1 (1)	0.579
Repeated cesarean delivery	No. (%)	58 (50.0)	51 (47.7)	0.789
Gestational age (weeks)	Mean (SD)	37.0 (0.8)	36.9 (0.9)	0.355
Details of surgery and anesthesia				
Duration of surgery (min)	Mean (SD)	67.8 (31.1)	65.4 (22.3)	0.512
Emergency (%)	No. (%)	14 (12.1)	11 (10.2)	0.655
Intrathecal injection of morphine (%)	No. (%)	99 (85.3)	88 (81.5)	0.437
Epidural analgesia	No. (%)	8 (6.9)	12 (11.1)	0.35
General anesthesia	No. (%)	6 (5.2)	3 (2.8)	0.501
Vertical incision	No. (%)	71 (61.2)	65 (60.2)	0.812
Maternal adverse reactions				
Pain at rest on POD1	(%)	24 (35.3)	22 (29.7)	0.479
on POD2	(%)	12 (20.3)	11 (16.2)	0.544
Nausea on POD1	(%)	14 (15.9)	13 (15.7)	0.965
Vomiting on POD1	(%)	10 (11.4)	5 (6.1)	0.226
Pruritus on POD1	(%)	24 (27.3)	29 (35.8)	0.232
Sedation on POD1	(%)	19 (22.9)	25 (30.9)	0.249

SD, standard deviation; IQR, interquartile range; POD, postoperative day.

Table 7 shows univariate analyses of factors associated with any persistent pain at 3 months. Among the 19 factors analyzed, the factors with p<0.1 were weight (p = 0.008), BMI (p = 0.033), emergency surgery (p = 0.037), intrathecal morphine (p = 0.016) and general anesthesia (p = 0.047). These five factors were retained for multivariate analyses. After final multivariate analyses, weight (p = 0.011) and intrathecal morphine (p = 0.023) remained determinant factors associated with persistent pain at 3 months (Table 8).

**Table 7:** Univariate analysis of factors associated with persistent pain at 3 months.

	OR (95% CI)	p
Maternal demographics		
Age	0.966 (0.907–1.030)	0.294
Height	0.964 (0.912–1.020)	0.202
Weight	0.953 (0.919–0.988)	0.008
Body mass index	0.903 (0.822–0.991)	0.033
Parity	0.958 (0.653–1.410)	0.826
Repeat cesarean delivery	0.963 (0.656–1.420)	0.848
Gestational age	1.130 (0.782–1.620)	0.525
Details of surgery and anesthesia		
Duration of surgery	0.999 (0.989–1.010)	0.890
Emergency	2.490 (1.060–5.870)	0.037
Intrathecal injection of morphine	0.405 (0.194–0.845)	0.016
Epidural analgesia	1.220 (0.466–3.220)	0.681
General anesthesia	2.970 (0.772–11.400)	0.113
Incision (vertical/transverse)	1.400 (0.772–2.540)	0.268
Maternal adverse reactions		
Pain at rest day on POD1	2.400 (0.689–8.380)	0.169
on POD2	0.882 (0.089–8.780)	0.915
Nausea on POD1	1.350 (0.573–3.180)	0.493
Vomiting on POD1	0.794 (0.241–2.620)	0.705
Pruritus on POD1	0.676 (0.324–1.410)	0.296
Sedation on POD1	0.728 (0.333–1.590)	0.427

POD, postoperative day; CI, confidence interval.

**Table 8:** Multivariate analyses of some important factors associated with persistent pain at 3 months.

	Adjusted OR (95% CI)	p
Maternal demographics		
Weight	0.955 (0.922–0.990)	0.011
Body mass index		>0.05
Details of surgery and anesthesia		
Emergency		>0.05
Intrathecal injection of morphine	0.424 (0.202–0.889)	0.023

OR, odds ratio; CI, confidence interval.

Table 9 illustrates the association among administration of morphine (i.t.), procedure type (elective/emergency) and type of anesthesia (regional/general). Emergency cases and those patients on general anesthesia had significantly less administration of morphine *via* the intrathecal route (p<0.001).

**Table 9:** Factors associated with intrathecal administration of morphine.

Variable	Morphine	Morphine	P
	(–)	(+)	
Elective	26	174	<0.001
Emergency	11	14	
Regional	30	186	<0.0001
General	7	2	
	Regional	General	
Elective	193	7	0.263
Emergency	23	2	

Table 10 emphasizes the association between anesthesia type and administration of intrathecal morphine. No patients with epidural anesthesia received intrathecal morphine.

**Table 10:** Association between type of anesthesia and intrathecal administration of bupivacaine, fentanyl and morphine.

Type of Anesthesia		Morphine		Total
		(-)	(+)	
	n	37	188	
Spinal only	No.	12	186	198
Spinal and epidural	No.	18	0	18
Spinal and general	No.	2	2	4
Spinal, general and epidural	No.	1	0	1
General only	No.	3	0	3
General and epidural	No.	1	0	1

SD, standard deviation.

We excluded patients with general anesthesia, and analyzed whether there were significant differences in persistent pain 3 months after delivery between patients with and without intrathecal morphine. We found that intrathecal injection of morphine significantly decreased the NRS of persistent pain (p = 0.0276).

We calculated the ratio of persistent pain at 3 months following delivery among patients who underwent spinal anesthesia only. As shown in Table 11, intrathecal morphine significantly reduced persistent pain, although there was no difference in the dose of intrathecal bupivacaine and fentanyl. By univariate analysis, the OR of intrathecal morphine towards reducing persistent pain was 0.263 with 0.0797 to 0.865 of 95% CI (p = 0.028).

**Table 11:** Effect of intrathecal administration of morphine on persistent pain at 3 months following delivery among patients who had spinal anesthesia only.

Variables		Morphine		p value
		(–)	(+)	
Persistent pain (+)	No. (%)	7 (58.3)	50 (26.9)	0.0416
Total	No.	12	186	
Dose of intrathecal Bupivacaine (ug/kg)	Mean (SD)	199 (32)	195 (26)	0.442
Fentanyl (ng/mL)	Mean (SD)	166 (27)	162 (22)	0.442
Morphine (ng/mL)	Mean (SD)	0	1624 (216)	<0.0001

## Discussion

### Key findings

We investigated the prevalence of chronic pain after cesarean section prospectively in a single institution in Japan. Among 225 patients who returned our questionnaires, 69 (30.7%) of patients complained of persistent pain 3 months after surgery, but no patient required medication. This prevalence was significantly high compared with that noted in studies evaluating chronic pain [2, 3, 5]. Multivariate analyses identified reduced weight (p = 0.011) and no intrathecal administration of morphine (p = 0.023) to be associated with persistent pain at 3 months. The adjusted OR of intrathecal administration of morphine to reduce persistent pain was 0.424, suggesting that intrathecal administration of morphine reduced the prevalence of pain by ≤50%. Indeed, 45.9% of patients who did not have intrathecal administration of morphine complained of persistent pain, whereas 27.7% of patients who had intrathecal administration of morphine complained of persistent pain. Also, 51.6% of patients had an abnormal wound sensation, suggesting the development of neuropathic pain. We also found that 6% of patients with abnormal wound sensation required medication. In contrast, no patients with persistent pain required medication.

### Relationship to previous studies

Severe acute postoperative pain has been highlighted as a factor that increases the risk of chronic pain [11]. With respect to cesarean section, conflicting data have been reported, although our study did not show an association between severe acute pain on POD1 and chronic pain at 3 months. Eisenach et al [2] evaluated 1288 patients and found that the severity of acute pain response to childbirth (and not cesarean delivery) can be used to predict persistent morbidity. They showed that the prevalence of severe acute pain <36 h *post partum* was 10.9%. In our study, 8.3% of patients complained of pain at rest on POD1. Although the prevalence of severe acute pain was comparable, its prevalence at <24 h was not associated with persistent pain.

Chronic pain after cesarean delivery was 30.1%, which was higher than that of studies in other countries: it also has wide variations, mainly because ethnicities and sampling methods are different [11]. In 2004, Nikolajsen et al. were first to report the prevalence of 12.3% for chronic pain at 3 months after cesarean section in a population in Denmark [3]. Kainu et al. reported a prevalence of chronic pain (18%) one year after cesarean section in Finland, which was higher than that occurring after vaginal birth (10%) [12]. Sng et al. reported a prevalence of 9.2% for wound-scar pain at 3 months after cesarean section done under spinal anesthesia in Singapore [5]. Liu et al. evaluated 426 patients in Australia, and reported that persistent pain at 2 months was 14.6%, but was reduced to 4.2% at 12 months. In our study, over half of all patients had abnormal wound sensations at 3 months post cesarean section, seemed to be as serious as the high incidence of chronic pain.

One factor that might have influenced the high prevalence of chronic pain in our study was the high mean age (35.4) of mothers, obviously meaning advanced reproductive age with increased pregnancy complications and chronic pain. According to the Japanese Ministry of Health, Labor and Welfare, the average age of first-time mothers has been increasing; it was 29.7% in 2009 and 30.6% in 2015, while the average age is relatively higher in Tokyo compared to other cities. The most frequent age group of first-time mothers is 30 to 34 in Japan. Because our institution is a university hospital in Tokyo, and 50.7% of patients had repeated cesarean delivery, the mean age tended to be over 35. Andreea et al reported that 27.4% of all women who died of pregnancy complications during 2006 to 2010 in the Unites States were aged 35 years or older [13]. In a study evaluating the prevalence and characteristics of chronic musculoskeletal pain in Japan, Nakamura et al reported that the prevalence of chronic musculoskeletal pain was significantly higher among women than men, higher in large cities, and highest for women in their 40s (18.6%), followed by those in their 30s (18.3%) and 50s (17%) [14]. We speculate that university hospitals tend to have patients with increased risk of pregnancy complications, in that the risk of chronic pain could be increased. Further studies are necessary.

Known risk factors associated with the development of chronic pain after cesarean delivery include poorly controlled acute pain [2,5], type of anesthetic used [3], history of cesarean delivery, and procedure type (emergency or elective)[15]. In our study, although univariate analyses revealed five factors (weight, BMI, emergency surgery, intrathecal administration of morphine and general anesthesia), multivariate analyses revealed weight and intrathecal administration of morphine as determining factors associated with persistent pain at 3 months. At our institution, as shown in Table 9, emergency cases received significantly less morphine *via* the intrathecal route, possibly due to the time involved in preparing a small dose of morphine. Patients who had general anesthesia usually did not receive spinal anesthesia and morphine *via* the intrathecal route. (Two cases had spinal anesthesia and general anesthesia, but the former was insufficient and they were switched to general anesthesia). After excluding confounding factors, neither procedure type nor type of anesthesia had an effect, but instead intrathecal administration of morphine was the defining factor for chronic pain.

It may reflect Japanese women's cultural perspectives, i.e., that although 30.7% of patients complained of persistent pain and 4.4% patients complained of pain that limited daily function, no patient required medication. Doering et al reported that most women manage labor pain without pharmacological interventions in Japan, and that Japanese women's cultural perspectives and passive attitudes were found to influence the decision-making process concerning pain relief [16]. According to the survey of Nakamura et al [14], among patients with chronic musculoskeletal pain, only 19% of patients visited a medical institution, and folk remedies accounted for almost the same proportion of treatment. Because we performed a letter-based interviews, it might have been easier to complain about chronic pain without visiting our hospital.

### Significance and implications

The present study showed a novel finding: absence of intrathecal administration of morphine increases chronic pain after cesarean section. This result reflected the fact that anesthetic management for cesarean section in our institution is not standardized, and that some patients did not receive morphine *via* the intrathecal route, which is the “gold standard” for pain management with cesarean section [1]. Known adverse effects of intrathecal morphine have been widely reported, and include pruritus, nausea and vomiting, urinary retention, and early or delayed respiratory depression. Respiratory depression was the worst life-threatening adverse effect, so nurses were given additional information according to the report by Kato et al. [17]. Use of intrathecal morphine for cesarean section is being developed by anesthesiologists during the study at our institution. The morphine dose given *via* the intrathecal route was 0.1 mg, and respiratory depression was not reported. We now routinely use intrathecal administration of morphine for cesarean section.

### Strengths and limitations

This prospective study had several limitations. First, collecting information on maternal adverse reactions (nausea, vomiting, pruritus, sedation) on POD1 and 2 was suboptimal. These data were obtained from nursing records, so this *hiatus* might have reflected poor observation of patients, and might have resulted in poor management of their pain. However, the median score of the PHPS as assessed by anesthesiologists was 2, suggesting that acute pain was managed to some degree.

Second, we did not standardize use of morphine given *via* the intrathecal route in the study period, and this type of administration was limited to anesthesiologists who favored it. This factor may have been because patients given morphine *via* the intrathecal route tend to be cared for by specific anesthesiologists.

## Conclusion

We found that 30.7% of patients complained of persistent pain after cesarean section. Although no effect on acute pain was observed, intrathecal administration of morphine significantly decreased the prevalence of chronic pain.
